# Reading without phonology: ERP evidence from skilled deaf readers of Spanish

**DOI:** 10.1038/s41598-021-84490-5

**Published:** 2021-03-04

**Authors:** Brendan Costello, Sendy Caffarra, Noemi Fariña, Jon Andoni Duñabeitia, Manuel Carreiras

**Affiliations:** 1 Basque Center on Cognition, Brain and Language, Paseo Mikeletegi, 69, 20009 Donostia-San Sebstián, Spain; 2 Division of Developmental-Behavioral Pediatrics, Stanford University School of Medicine, Stanford University, Stanford, CA USA; 3 Stanford University Graduate School of Education, Stanford, CA USA; 4 Departamento de Psicología de la Educación y Psicobiología, Facultad de Educación, Universidad Internacional de La Rioja, Logroño, Spain; 5 Centro de Ciencia Cognitiva – C3, Universidad Nebrija, Madrid, Spain; 6 Department of Language and Culture, The Arctic University of Norway, Tromsö, Norway; 7 Departamento de Lengua Vasca y Comunicación, UPV/EHU, Bilbao, Spain; 8 Basque Foundation for Science, Bilbao, Spain

**Keywords:** Language, Reading

## Abstract

Reading typically involves phonological mediation, especially for transparent orthographies with a regular letter to sound correspondence. In this study we ask whether phonological coding is a necessary part of the reading process by examining prelingually deaf individuals who are skilled readers of Spanish. We conducted two EEG experiments exploiting the pseudohomophone effect, in which nonwords that sound like words elicit phonological encoding during reading. The first, a semantic categorization task with masked priming, resulted in modulation of the N250 by pseudohomophone primes in hearing but not in deaf readers. The second, a lexical decision task, confirmed the pattern: hearing readers had increased errors and an attenuated N400 response for pseudohomophones compared to control pseudowords, whereas deaf readers did not treat pseudohomophones any differently from pseudowords, either behaviourally or in the ERP response. These results offer converging evidence that skilled deaf readers do not rely on phonological coding during visual word recognition. Furthermore, the finding demonstrates that reading can take place in the absence of phonological activation, and we speculate about the alternative mechanisms that allow these deaf individuals to read competently.

## Introduction

Learning to read is a transformative experience for socio-economic mobility and for our cognitive development^1,2^. Knowing how to read is closely tied to our knowledge of the language we are reading. The written form is a visual representation of auditory words, and learning to read an alphabetic script typically involves associating letters with sounds. During reading acquisition, phonological processes are the first to develop^3–5^ and lexical access involves phonological mediation^6–8^; reading deficits, such as dyslexia, are associated with poor phonological awareness^9^. The extent to which phonological representations play a role in reading depends on reading proficiency but also on the transparency of the writing system^10–12^. More proficient readers make greater use of strategies that bypass phonology by recognizing orthographic units or even whole words^13,14^. Transparent orthographies, in which there is a regular correspondence between graphemes and phonemes, favour phonological coding during word recognition^15^ and may impact the acquisition of syntactic rules^16^. In contrast, in opaque orthographies, readers can process printed words by using alternative strategies, such as relying on the visual-orthographic structure. In a language with a transparent orthography, like Spanish, phonological codes are automatically accessed during reading^17,18^. In this study, we ask whether phonological processing is required for word identification in a transparent orthography. To do this, we examine a population that reads without access to the phonological forms of words, namely, deaf individuals.

A variety of factors, including visuo-perceptual processing and reading experience, impact reading outcomes^19^ but in the case of deaf readers, their lack of audition severely limits their access to phonology. Consequently, the connection between the written word and the sound-based form that it represents has led various authors and educators to highlight the importance of phonological coding and awareness for reading skills in this population^20–22^. Many studies that look at the role of phonology in deaf readers do so through meta-phonological tasks and/or phonological awareness tasks that require explicit phonological judgments, such as rhyme detection^23–29^. Since our aim is to determine whether phonology is automatically activated in word reading, we wish to avoid task-driven effects that might activate phonological representations: we focus on implicit reading tasks that do not involve making decisions about the phonological form of the word.

Despite the evidence of phonological encoding from studies with explicit tasks, phonology seems to be much less important for deaf readers^9,30^. Reading ability in English of primary school children has been linked to language ability but not phonemic awareness^31^. A major study of over 200 secondary school deaf readers (aged between 11 and 16 years old) of four different languages (with transparent and opaque orthographies) found that syntactic rather than phonological knowledge characterized skilled readers from those less skilled^16^. In a meta-analysis of 57 studies looking at reading in deaf individuals, phonological awareness predicted only 11% of the variance in reading ability; in contrast, overall language ability (either spoken or signed) predicted up to 35%^32^. Further support comes from specific studies that have found no behavioural evidence of phonological encoding in deaf readers^33^. Skilled deaf readers of French activated visual, orthographic and semantic codes during reading, but not phonological codes^34^. In an eye tracking study of the phonological and orthographic preview benefit in the parafovea in readers of English, both hearing and deaf readers benefited from orthographic coding in parafoveal vision but only the hearing group benefited from phonological coding^35^.

Much of the previous work on deaf readers^34–36^ has been conducted in languages with opaque orthographies, particularly English. Since orthographic depth can modulate the role of phonological coding during reading^4,10–12^, the absence of phonological coding in deaf readers of languages like English^31^ or Hebrew^16^ may be due to the opaque orthography. With a transparent orthography, the properties of the orthographic representation lead us to expect the engagement of phonological encoding: a study of deaf readers of Spanish found evidence of phonological coding^37^. Critically, the deaf readers in that study had a broad range of reading abilities. In this study we focus on the minority of deaf readers who have good reading skills. For those deaf individuals who read a transparent orthography well, what role does phonology play?

The present study investigates the involvement of phonological processes during visual word recognition in deaf skilled readers of a transparent language (Spanish) using tasks that do not explicitly require phonological analysis (a semantic categorization task and a lexical decision task) while measuring behavioural responses and electrophysiological activity (EEG), and to compare the group with hearing readers. EEG provides sufficient temporal precision to capture the time course of sub-lexical processes (i.e., early and late effects). An important feature of this study is that deaf participants are skilled readers with reading levels comparable to hearing readers of the same age. We carried out two experiments, each looking at different aspects of the word recognition process: the first focuses on sub-lexical processing during word recognition to look for evidence of phonological activation before lexical access takes place; the second experiment examines lexical analysis and the possible role of phonological coding during lexical access. We exploit the pseudohomophone effect, based on pseudowords that sound like real words, one of the strongest indicators of phonological processing in visual word recognition^38–41^. Our goal is to discover whether these proficient deaf readers activate phonological representations and to what extent they differ from hearing readers. More broadly, we inquire how central phonology is for proficient reading in a language with a transparent orthography.

## Results

### Experiment 1: semantic categorization task

In Experiment 1 we tested the role of phonological processing with a go/no-go semantic categorization task (identifying animals) with masked priming. In the critical, no-go trials, primes were pseudowords and pseudohomophones of the target word. Masked priming has revealed early phonological effects in visual word recognition: the N250 component, with a broad scalp distribution, reflects sub-lexical processing that involves associating orthographic and phonological representations, and takes place before lexical access has occurred^42^. Compared with control pseudoword primes, pseudohomophone primes modulate the N250 component^43^ and we expect this effect for the hearing readers. If deaf readers activate phonological codes, we expect a comparable modulation; conversely, if they do not activate phonological codes the response should be similar for both types of prime (pseudohomophones and control pseudowords).

### Behavioural

In the semantic categorization task all participants showed high levels of accuracy, pressing the button response when the animal names were presented as target (range 80–100%, mean 99.0%, SD 3.43). When the animal names were presented as prime (for just 50 ms), the response rate was close to zero (mean 1.38%, SD 2.53) indicating that the primes were successfully masked. There were only few false alarms in the experimental trials (pseudohomophone primes: mean 2.56%, SD 2.68; pseudoword primes: mean 2.0%, SD 1.98) suggesting that participants paid attention to the content of the stimuli.

### ERPs

**Figure 1:**
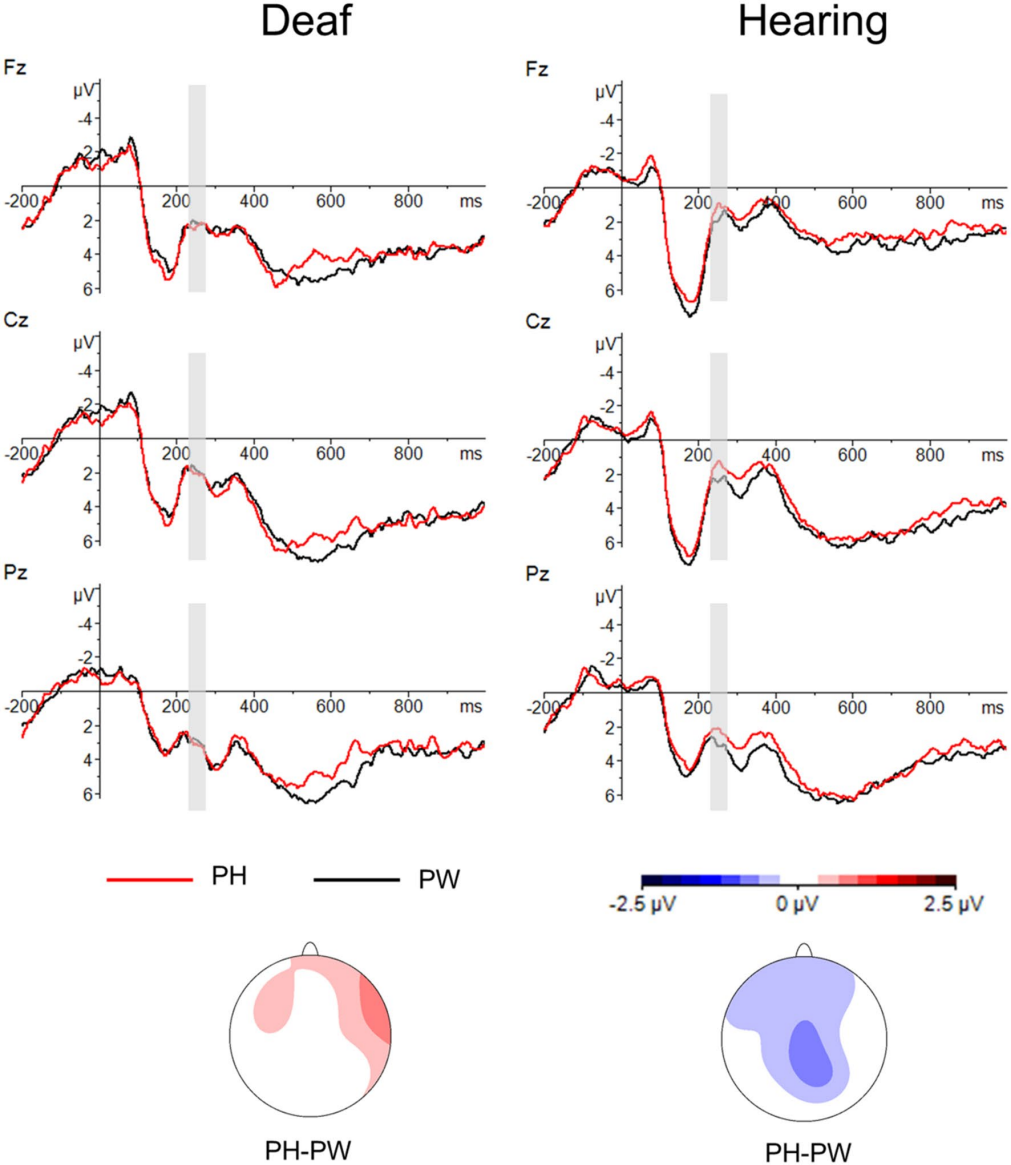
ERP results for Experiment 1 (semantic categorization task). Top: grand average ERP waveforms for deaf (left) and hearing (right) groups. Midline electrodes (Fz, Cz, Pz) are displayed. ERPs in response to the target words are compared for pseudohomophone and pseudoword primes. Bottom: N250 topographical maps created by subtracting the average voltage of pseudoword prime condition from the average voltage of pseudohomophone prime condition. *PH* Pseudohomophones, *PW* Pseudowords.

As planned, we analysed the EEG signal of no-go trials in an early time window to examine the effect of Group (hearing, deaf), PrimeType (pseudohomophone, pseudoword) and topographic factors (see Methods) on the N250 component. Additionally, visual inspection of the grand average waveform revealed a divergence between prime types later on (see Fig. 1); analysis of a 500–700 ms window yielded a trend for a main effect of PrimeType that did not reach significance (see Supplementary Materials S1 for details).

### 230–270 ms

There was a significant interaction Group × PrimeType (*F*(1,38) = 4.10, *p* = 0.049, ŋ^2^ = 0.097; see Fig. 1) suggesting that in the hearing group targets preceded by pseudohomophones elicited a greater negativity as compared to targets preceded by pseudowords (*t*(19) = 2.55, *p* = 0.039). The two conditions did not differ in the deaf group (*t*(19) = 0.79, *p* = 0.439). No other significant effect was found (Group: *F*(1,38) = 0.14, *p* = 0.712, ŋ^2^ = 0.004; PrimeType: *F*(1,38) = 0.89, *p* = 0.351, ŋ^2^ = 0.023).

### Experiment 2: Lexical decision task

Experiment 2 tested phonological processing in a lexical decision task with words and two types of nonwords: pseudohomophones and control pseudowords. Behaviourally, the pseudohomophone effect shows up as slower reaction times and/or more errors for pseudohomophones compared to control pseudowords^40^. In electrophysiological measures, the N400, a component with a typically centro-parietal distribution, has been associated with semantic incongruence and semantic processing in written word recognition^44^. Reading nonwords compared with words elicits a more negative amplitude around 400ms^45^. However, phonology can modulate the ERP response^46^: nonwords that sound like real words—pseudohomophones—attenuate the N400 amplitude^38,47,48^. The reduced N400 effect for pseudohomophones suggests that the phonological representation facilitates semantic integration in visual word recognition^49^. We expect to replicate the results from previous experiments in the hearing group: a pseudohomophone effect in the behavioural and ERP responses. If deaf skilled readers use phonological encoding during lexical access, we expect similar results for this group also. Conversely, if deaf readers are not sensitive to phonological codes, we expect their responses to pseudohomophones not to differ from those of pseudowords, with similar error rates and reaction times, and a similar N400 amplitude.

### Behavioural measures

#### Error rates

**Figure 2:**
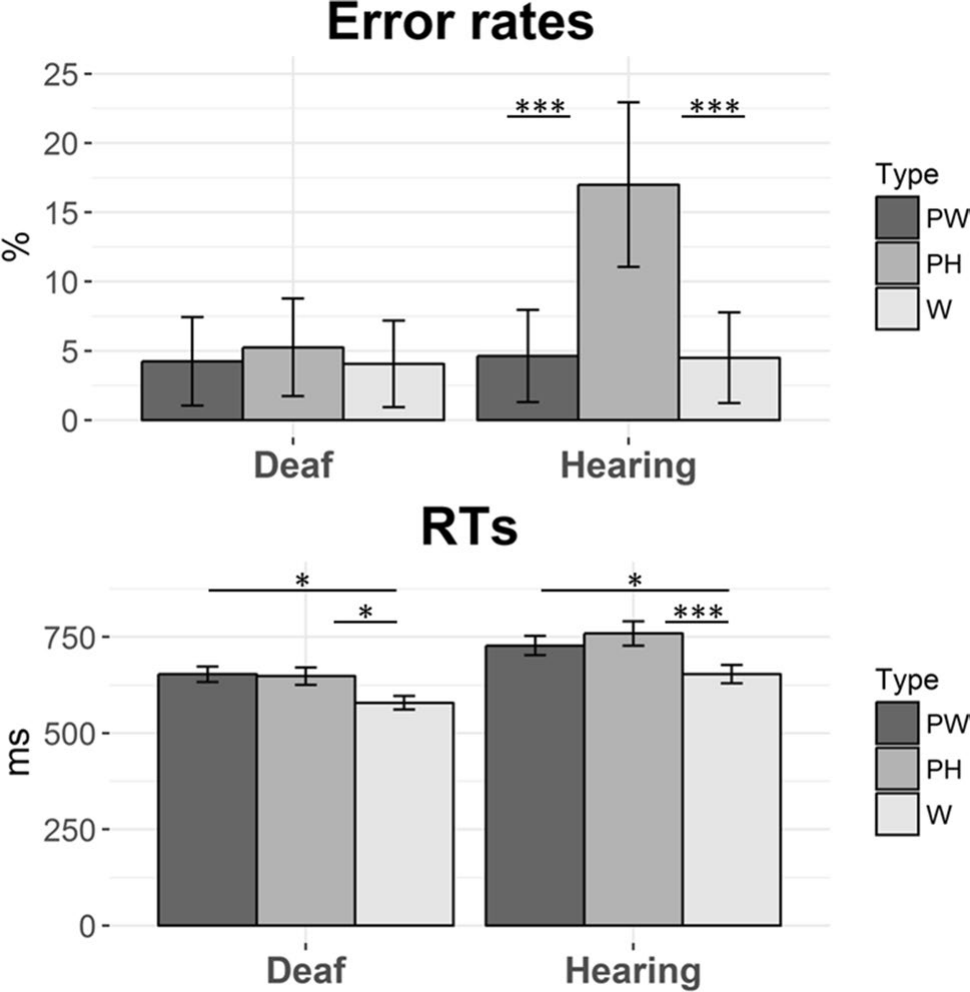
Mean error rates and RTs for the deaf and the hearing groups in Experiment 2 (lexical decision task). Error bars correspond to one standard error. *PW* Pseudowords, *PH* Pseudohomophones, *W* Words.

The deaf group was more accurate than the hearing group (*F*_*1*_(1, 38) = 10.05, *p* = 0.003; *F*_*2*_(1, 237) = 30.37, *p* < 0.001, ŋ^2^ = 0.209; see Table 1 and Fig. 2). The effect of WordType (word, pseudohomophone, pseudoword) was also significant (*F*_*1*_(2, 76) = 21.70, *p* < 0.001; *F*_*2*_(2, 237) = 16.00, *p* < 0.001, ŋ^2^ = 0.363), indicating that participants made the highest number of errors with pseudohomophones as compared to the other two types of stimuli (words: *t*(117) = 4.46, *p* < 0.001; pseudowords: *t*(117) = 4.36, *p* < 0.001). The interaction Group × WordType (*F*_*1*_(2, 76) = 15.24, *p* < 0.001; *F*_*2*_(2, 237) = 24.22, *p* < 0.001, ŋ^2^ = 0.286) showed that this difference was present in the hearing group (pseudohomophones vs. words: *t*(114) = 6.57, *p* < 0.001; pseudohomophones vs. pseudowords: *t*(114) = 6.50, *p* < 0.001), but not in the deaf group (pseudohomophones vs. words: *t*(114) = 0.62, *p* = 0.950; pseudohomophones vs. pseudowords: *t*(114) = 0.53, *p* = 0.950).

**Table 1:** Average and SDs of error rates and RTs for each condition for Experiment 2 (lexical decision task).

	Deaf		Hearing	
	mean	SD	mean	SD
**Error Rates**				
Pseudowords	4.25	20.19	4.63	21.02
Pseudohomophones	5.25	22.32	17.00	37.59
Words	4.06	19.75	4.50	20.74
**RTs**				
Pseudowords	653	126	728	157
Pseudohomophones	648	142	759	202
Words	579	112	653	151

#### RTs

The deaf group was faster than the hearing group (*F*_*1*_(1, 38) = 10.39, *p* = 0.003; *F*_*2*_(1, 237) = 424.59, *p* < 0.001, ŋ^2^ = 0.215; see Table 1 and Fig. 2). A main effect of WordType (*F*_*1*_(2, 76) = 93.22, *p* < 0.001; *F*_*2*_(2, 237) = 90.53, *p* < 0.001, ŋ^2^ = 0.710) indicated that responses to the words were faster as compared to the two types of nonwords (pseudohomophones: *t*(117) = 4.08, *p* < 0.001; pseudowords: *t*(117) = 3.34, *p* = 0.002). Importantly, this effect interacted with Group (*F*_*1*_(2, 76) = 6.57, *p* = 0.014; *F*_*2*_(2, 237) = 8.03, *p* = 0.023, ŋ^2^ = 0.147), suggesting that the difference between pseudohomophones and words was statistically more evident in the hearing group as compared to the deaf group (hearing: *t*(114) = 3.99, *p* < 0.001; deaf: *t*(114) = 2.42, *p* = 0.024), while the difference between pseudowords and words was similarly present in both groups (hearing: *t*(114) = 2.63, *p* = 0.018; deaf: *t*(114) = 2.62, *p* = 0.018). These behavioural results were confirmed when by-item and by-participant variability were accounted for by mixed linear models analyses (see Supplementary Materials S2).

### ERPs

#### 300–500 ms

**Figure 3:**
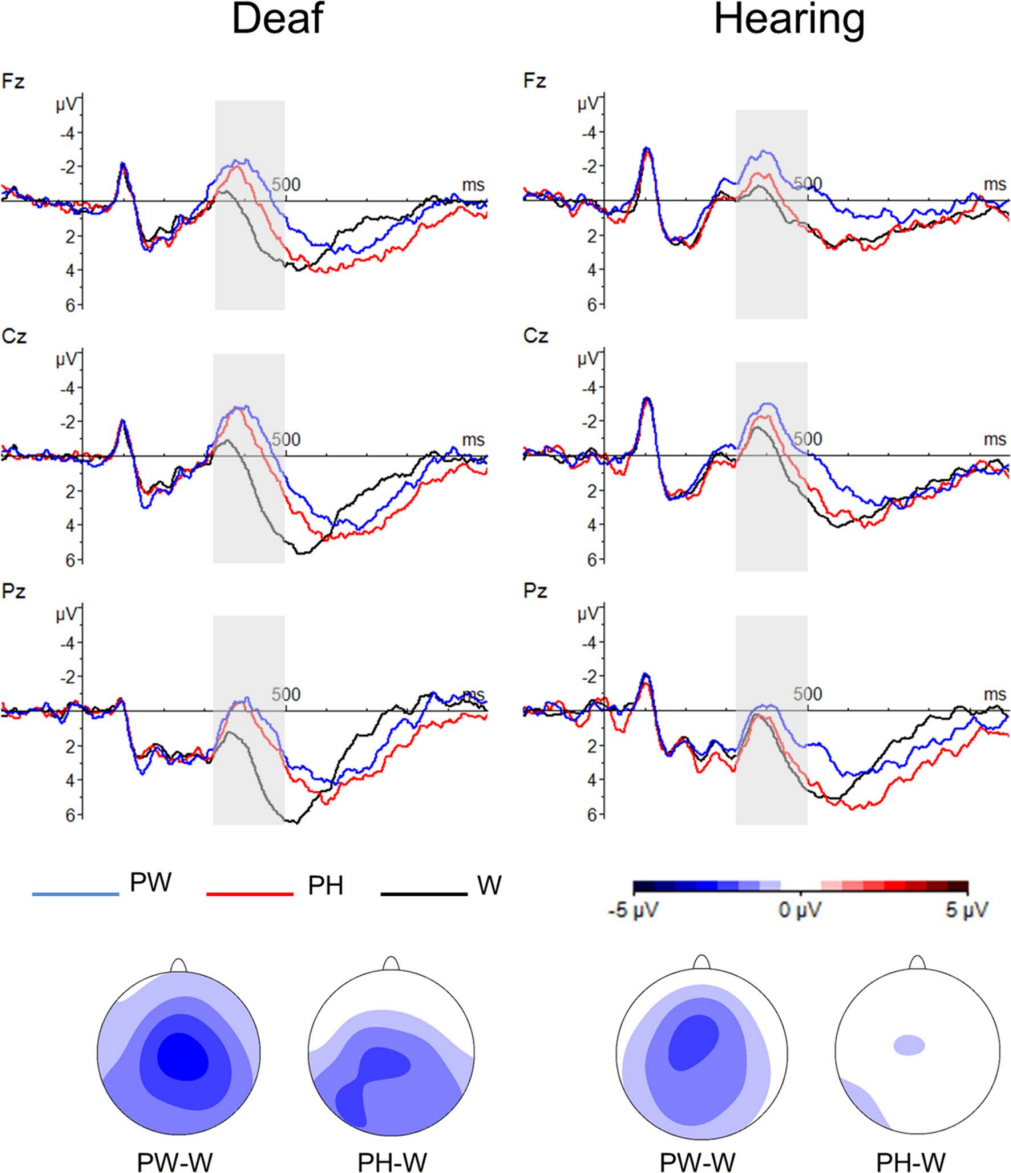
ERP results for Experiment 2 (lexical decision task). Top: grand average ERP waveforms for deaf (left) and hearing (right) groups. Midline electrodes (Fz, Cz, Pz) are displayed. Bottom: N400 topographical maps for 300–500 ms created by subtracting the average voltage of words from the average voltage of pseudowords, and the average voltage of words from the average voltage of pseudohomophones. *PW* Pseudowords, *PH* Pseudohomophones, *W* Words.

There was a main effect of WordType (*F*(2, 76) = 18.31, *p* < 0.001, ŋ^2^ = 0.325, see Fig. 3), suggesting that the greatest negativity was elicited by the pseudowords, which were followed by the pseudohomophones and the words (pseudowords vs. pseudohomophones: *t*(39) = 2.88, *p* = 0.007; pseudohomophones vs. words: *t*(39) = 3.44, *p* = 0.003; pseudowords vs. words: *t*(39) = 6.29, *p* < 0.001). The interactions between WordType and the topographical factors (Type × Anteriority: *F*(4, 152) = 9.89, *p* < 0.001, ŋ^2^ = 0.206; WordType × Laterality: *F*(4, 152) = 3.62, *p* = 0.010, ŋ^2^ = 0.087; WordType × Anteriority × Laterality: *F*(8, 304) = 2.10, *p* = 0.057, ŋ^2^ = 0.052) indicated that these differences were more evident over central right electrodes (all *t*s > 2.60), which is compatible with the N400 distribution. In addition, the factor WordType interacted with Group (Group x WordType: *F*(2, 76) = 3.47, *p* = 0.036, ŋ^2^ = 0.084; Group × WordType × Anteriority: *F*(4, 152) = 3.10, *p* = 0.037, ŋ^2^ = 0.075), indicating different N400 effects for deaf and hearing participants. While in the hearing group only pseudowords elicited a greater N400 as compared to words and pseudohomophones (pseudowords vs. words: *t*(19) = 3.93, *p* = 0.003; pseudowords vs. pseudohomophones: *t*(19) = 3.96, *p* = 0.003; pseudohomophones vs. words: *t*(19) = 0.84, *p* = 0.413), in the deaf group both pseudowords and pseudohomophones elicited a greater N400 as compared to words (pseudowords vs. words: *t*(19) = 5.09, *p* < 0.001; pseudohomophones vs. words: *t*(19) = 4.12, *p* = 0.003; pseudowords vs. pseudohomophones: *t*(19) = 1.09, *p* = 0.413). These differences were more evident over central and posterior sites (hearing: all *t*s > 3.00; deaf: all *t*s > 4.00). Similar ERP results were obtained using the same amount of items for each level of the factor WordType (see Supplementary Materials S3).

## Discussion

The pseudohomophone effect in hearing readers provides clear evidence of phonological activation in word reading. In experiment 1, pseudohomophone primes modulated hearing readers’ N250 response, indicating that phonological representations participate in sub-lexical processing. In experiment 2, hearing readers made significantly more errors in classifying pseudohomophones as nonwords compared to control pseudowords, and their ERP response also showed a reduced N400 effect for pseudohomophones compared to control pseudowords, replicating previous studies that showed that phonological information interferes in the process of lexical decision^38,39,41^.

In contrast to the hearing readers, the skilled deaf readers in this study showed no difficulty in classifying pseudohomophones as nonwords: they showed no difference in error rates (or reaction times) or in the N400 response for pseudohomophones and control pseudowords. These deaf readers treated pseudohomophones as pseudowords; the fact that they sound like real words created no interference. We take this as evidence that these skilled deaf readers do not need to activate phonological coding during word reading. Even so, phonological coding might occur but not be apparent (because another, more effective mechanism achieves the word recognition process independently). Two pieces of evidence suggest that this is not the case. Firstly, in the semantic categorization task, pseudohomophones did not modulate deaf readers’ N250, suggesting that even at the sub-lexical level phonological coding is not activated. Secondly, in the lexical decision task, deaf readers responded significantly faster than hearing readers across all conditions. Previous studies have reported faster reaction times in word reading tasks for deaf compared to hearing readers^20,50,51^. Deaf individuals show enhanced reactivity in simple visual detection tasks^52^ and this may explain part of this advantage. An additional contributing factor for the discrepancy between deaf and hearing readers in word detection tasks may be precisely that phonological competitors are not activated for deaf readers, making word recognition faster^51,53^. This proposal fits with the general trend for faster, more proficient reading to rely less on phonological encoding and more on orthographic chunking^13,54^.

These results provide converging behavioural and electrophysiological evidence that these skilled deaf readers read words without activating phonology, as has already been shown behaviourally for other languages with transparent orthographies, such as German or Turkish^19^. This finding casts doubt on the assumption that deaf individuals need to access phonology in reading tasks to be competent readers^21^. Although some studies show a correlation between phonology and reading skills in deaf individuals^24,26,55,56^, our finding adds neuroimaging evidence to the body of studies that question the importance of phonological coding for proficient reading in the prelingually deaf^16,19,20,30–32,34,35,57–59^. Central to this issue is the type of deaf reader: this study has focused on highly skilled deaf readers, that is, individuals who have overcome the condition of deafness to acquire competent reading skills. Other deaf readers may have different processing mechanisms; a previous study with deaf readers of Spanish who were not highly skilled did find evidence of phonological encoding^37^.

This leads to an important caveat for our finding in terms of what it tells us about deaf readers: our electrophysiological data support the claim that highly skilled deaf readers do not need to—and in fact do not—automatically activate phonological encoding. We have examined the end state of the process of acquiring reading but make no claims about the process of learning to read. For hearing learning readers, phonology seems to play an important role^14^. The evidence for phonological coding in deaf children learning to read is mixed^60,61^ and more work is required to understand what phonology does and does not contribute to reading for different deaf individuals, but growing evidence suggests that the difference may lie at the sentence or discourse level rather than the lexical level^19^. This caveat limits the implications of this study for educational practice. Explicit training of phonological awareness may not help deaf individuals to achieve high reading proficiency^22,24,31^. Deaf readers do not need phonology during automatic stages of reading processing, suggesting that these readers avail of alternative strategies. One possibility is a visual, purely orthographic representation, supported by neuroimaging evidence that deaf readers have finely tuned orthographic representations but weak phonological sensitivity in the reading network^62^; another is mediation of the written word through the sub-lexical units of sign language^9,63^. Future studies should investigate how these processes support skilled deaf readers in acquiring a high level of literacy and how these mechanisms could be exploited and fostered to improve reading skills in deaf students.

To conclude, our findings tell us something important about reading in general. Contrary to previous claims^17,18^, phonological mediation is not necessarily an automatic part of reading a language with a transparent orthography. Skilled deaf readers in the current study did not activate phonological codes, showing that phonological access is not necessary for competent reading in Spanish. Even though phonological knowledge may facilitate learning to read a transparent orthography, the present results demonstrate that this does not mean that phonology is required during skilled reading.

## Methods

### Experiment 1: semantic categorization task

#### Participants

Twenty adult severely (70–90 dB) to profoundly (> 90 dB) deaf adults (14 females; age range 23–45 years old; mean 33; SD 7) participated in the study. All participants were born deaf or lost their hearing before the age of 3 (i.e., prelingual deafness); none had cochlear implants. They learned Spanish Sign Language before the age of 10 and used it as the main language for communication. Most of them learned to read at an early age, at school, except two individuals who learned to read after the age of 16. Twenty hearing Spanish readers (10 females; age range = 20–42 years old; mean 29; SD 6) were included as a control group. All deaf participants and hearing controls were skilled readers in Spanish (i.e., with performances above the 75th centile of the ECL-2 Test^64^) and the two groups did not differ in non-verbal intelligence, Spanish reading comprehension, and Spanish vocabulary size (see Table 2). Before taking part in the experiment all participants signed an informed consent form that was approved by the BCBL Ethics Committee. The experiment was performed following the BCBL Ethics Committee’s guidelines and regulations.

**Table 2:** Characteristics of the experimental and control groups.

	Deaf		Hearing		*t*	*p*
	mean (SD)	range	mean (SD)	range		
Non-verbal Intelligence	84.15 (9.74)	75–99	86.40 (9.88)	75–99	0.73	0.47
Reading comprehension	88.75 (8.81)	75–99	92.90 (7.86)	75–99	1.57	0.12
Vocabulary size	94.58 (5.93)	78.33–100	90.96 (12.93)	51.66–100	1.13	0.26

Non-verbal intelligence was measured with the Raven Progressive Matrices test^69^, where participants had to identify the missing item of a sequence of figures. Spanish reading comprehension was assessed through the ECL-2^64^, a standardized reading test consisting of five short paragraphs followed by a total of 27 multiple-choice questions evaluating word meaning, synonyms, antonyms, sentence and text content. Vocabulary size was measured using the Spanish version of the LexTALE^70,71^, a lexical decision test consisting of 60 real words and 30 nonwords providing a good estimate of language knowledge^72^. All scores are percentages. Results of two-tailed *t*-tests are reported for each group comparison.

### Materials

The experimental targets were 80 Spanish words (4–6 letters long; mean number of letters 5.29; mean log word frequency 3.96, range 2.61–4.65) selected from the EsPal database^65^. The targets were preceded by nonword primes that could be: (1) pseudohomophones (40 items), which were created by replacing one letter of the target word by another letter that corresponded to the same phoneme (e.g., the pseudohomophone *nobio* created from the base word *novio* [boyfriend]), and (2) pseudowords (40 items), in which one letter of the target was replaced by another letter that corresponded to a different sound (e.g., the pseudoword *notio* created from the same target word). Two lists were constructed such that each target word used to generate the nonwords was presented once in each list, either as a pseudohomophone or as a pseudoword.

In addition, we selected an additional 20 Spanish words (4–6 letters long) corresponding to animal names (mean log word frequency 3.49, range 2.26–4.50; mean number of letters: 4.50), each of which was paired with a Spanish word with the same number of letters (mean log word frequency 3.16, range 2.51–3.50; mean number of letters 4.50). Each word pair was not semantically related and was presented twice, once with the animal name as target and once with the animal name as prime. This was done to control for prime visibility.

Participants were randomly assigned to one of the two lists. In total, each participant completed 120 trials: 80 experimental trials with nonword-prime/word-target pairs (40 pseudohomophone primes and 40 pseudoword primes), 20 trials with word-prime/animal-target pairs, and 20 trials with animal-prime/word-target pairs.

### Procedure

**Figure 4:**
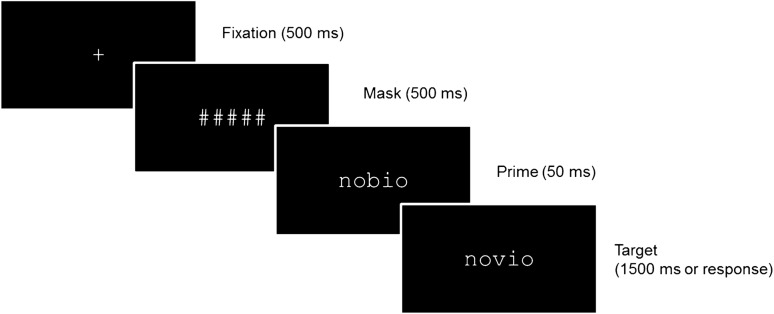
Schematic representation of a trial of Experiment 1 (semantic categorization task).

The experiment was run in a silent and dimly lit room using Presentation software (version 0.70). Each trial began with a fixation cross for 500 ms, followed by a forward mask consisting of a row of hash marks (#####) for 500 ms (the number of hash marks matched the length of the prime). Then, a prime appeared in lowercase (25-pt. Courier New) and remained on the screen for 50 ms. The prime was followed immediately by the target stimulus, also in lowercase. Stimuli subtended a visual angle of 1.9 to 2.9 degrees depending on the number of letters. The target remained on the screen for 1500 ms or until the participant’s response (see Fig. 4). Participants were instructed to press a button on the keyboard (space bar) only when they saw an animal name, and to respond as quickly and accurately as possible. The order of presentation of the stimuli was randomized for each participant.

### EEG recording and analysis

The electroencephalogram (EEG) was recorded with a 32-channel BrainAmp system (Brain Products GmbH) at a 500 Hz sampling rate. Scalp voltages were collected from 27 Ag/AgCl electrodes placed in an EasyCap recording cap (Fp1, Fp2, F7, F8, F3, F4, FC5, FC6, FC1, FC2, T7, T8, C3, C4, CP5, CP6, CP1, CP2, P3, P4, P7, P8, O1, O2, Fz, Cz, Pz). Two external electrodes were placed on the mastoids, with the right one as the online reference. An additional electrode at FCz served as ground, and four electrodes (two above and below the right eye, and two on the ocular canthi) recorded the electro-oculogram (EOG). Impedance was kept below 5KΩ for mastoids and scalp electrodes, and below 10KΩ for EOG electrodes. The EEG signal was re-referenced offline to the average activity of the two mastoids. A notch filter and a bandpass filter were applied (50 Hz, 0.01–30 Hz, 12 dB/octave). Ocular artifacts were corrected using Independent Component Analysis (ICA): the EEG of each subject was decomposed into independent components and those that explained the highest variance of EOG channels were identified and visually inspected before being removed from the original signal. Only trials corresponding to the experimental items were included in the analysis: all animal go trials and experimental trials with incorrect go-responses were excluded. For the remaining trials, EEG epochs of 1200 ms time-locked to the target presentation onset were obtained (including 200 ms pre-stimulus baseline). Residual artifacts exceeding ± 100 µV in amplitude were removed. Overall, 8.56% of trials were rejected, with no differences across experimental conditions (*F*(1,78) = 0.17, *p* = 0.68). The remaining clean epochs were averaged across conditions, with a baseline correction between – 200 and 0 ms.

We analysed the data using R^66^. A repeated-measure analysis of variance (ANOVA) was applied to the N250 time window, which was defined based on visual inspection (230–270 ms). An additional late time window was added to the analysis after visually inspecting the ERP grand averages (500–700 ms). The average activity of three contiguous electrodes was calculated resulting in nine clusters: Left Anterior (F3, F7, FC5), Left Central (C3, T7, CP5), Left Posterior (P3, P7, O1), Medial Anterior (Fp1, Fp2, Fz), Medial Central (FC1, FC2, Cz), Medial Posterior (CP1, CP2, Pz), Right Anterior (F4, F8, FC6), Right Central (C4, T8, CP6), Right Posterior (P4, P8, O2). To probe the scalp distribution of any effects, these clusters were included in the statistical analyses as different levels of two topographical factors: Anteriority (Anterior, Central, Posterior) and Laterality (Left, Middle, Right). Each ANOVA included the within-subject factor of PrimeType (Pseudohomophone, Pseudoword), the between-subject factor of Group (Deaf, Hearing), and the two topographical factors (Anteriority and Laterality). The Greenhouse–Geisser procedure was applied when the sphericity assumption was violated. Effects of topographical factors will be reported only when they interact with the experimental factors. Two-tailed *t*-tests were conducted as follow-up analyses and corrected for multiple comparisons^67^.

### Experiment 2: lexical decision task

#### Participants

The participants were the same as those in Experiment 1.

#### Materials

For word trials, we selected 80 Spanish words between four and six letters long (mean number of letters 5; average log frequency 3.57, range 3.01–3.87) from the EsPal database^65^. For nonword trials, 80 Spanish base words between four and six letters long (mean number of letters 5.29) with a similar frequency to the first set were also selected (mean log word frequency 3.96, range 2.61–4.65). These words were then used to create two types of nonwords: (1) pseudohomophones by replacing one letter by another letter that corresponded to the same phoneme (e.g., the pseudohomophone *javón* created from the base word *jabón* [soap]), and (2) pseudowords in which one letter was replaced by another letter that corresponded to a different sound (e.g., the pseudoword *jacón* created from the same base word). Two lists were constructed such that each base word used to generate the nonwords was presented once in each list, either as a pseudohomophone or as a pseudoword. Participants were randomly assigned to the two lists. In total, each participant completed 160 trials: 80 trials with words and 80 trials with nonwords (40 pseudohomophones and 40 pseudowords).

### Procedure

**Figure 5:**
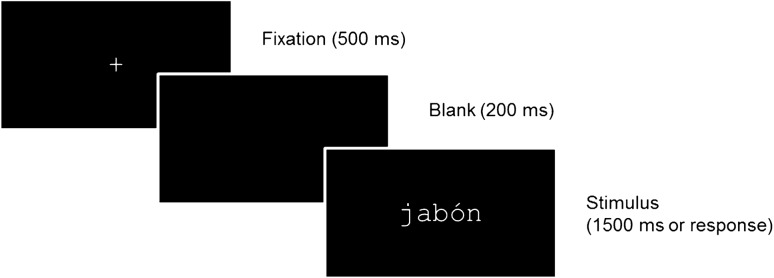
Schematic representation of a trial of Experiment 2 (lexical decision task).

The experiment was run in a silent and dimly lit room using Presentation software (version 0.70). Each trial began with a fixation cross at the centre of the screen for 500 ms, followed by a blank screen for 200 ms. Then, a stimulus (word or nonword) was presented in lowercase font (45-pt. Courier New, subtending a visual angle of 3.2 to 5.0 degrees) for 1500 ms or until the participant’s response (see Fig. 5). Participants were instructed to press one of two buttons on the keyboard (‘M’ and ‘Z’) to indicate whether the letter string was a word or a nonword. They were asked to respond as quickly and accurately as possible. The order of presentation of the stimuli was randomized for each participant and the two response buttons were counterbalanced for word and nonword responses. Participants completed eight practice trials before starting the experiment.

### EEG recording and analysis

The EEG recording and pre-processing were the same as in Experiment 1. ERPs time-locked to the onset of the stimulus presentation were obtained only for the trials followed by a correct behavioural response. Overall, the average number of rejected ERP trials was 11.17%. Statistical analyses were conducted on both behavioural and ERP data.

We analysed the data using R^66^ and the lme4 package^68^. For the behavioural data, we calculated error rates and reaction times (RTs). Trials with incorrect responses (5.94%) were excluded from RT analysis. RTs that were more than 2.5 SDs away from the mean of each condition and each participant were also excluded from the analysis of the response latencies (2.28%). A by-subject repeated-measures ANOVA was conducted on the error rates and the RTs including Group (Deaf, Hearing) as a between-subject factor, and WordType (Word, Pseudohomophone, Pseudoword) as a within subject factor. Similar ANOVAs were conducted by item, including Group as a within-item factor and WordType as a between-item factor.

For the EEG data, a repeated-measures ANOVA was conducted on the average ERP amplitude within the N400 time window (300–500 ms). This analysis included Group (Deaf, Hearing) as a between-subject factor and WordType (Word, Pseudohomophone, Pseudoword) as a within-subject factor. As in Experiment 1, two topographical factors were also added (Anteriority, Laterality). The Greenhouse–Geisser procedure was applied when the sphericity assumption was violated. Effects of topographical factors will be reported only when they interact with the experimental factors. In both behavioural and EEG analyses, two-tailed *t*-tests were conducted as follow-up analyses and corrected for multiple comparisons^67^.

## Supplementary Information

Supplementary Information.

## Data Availability

The datasets generated during and/or analysed during the current study are available from the corresponding author on reasonable request.
