# Supplementary material for: Reading without phonology: ERP evidence from skilled deaf readers of Spanish
Source: Sci Rep. 2021 Mar 4;11:5202. doi: 10.1038/s41598-021-84490-5 (PMC7933439; doi:10.1038/s41598-021-84490-5)
Supplement: Supplementary file 1 — Supplementary Information. [file 41598_2021_84490_MOESM1_ESM.pdf]

Costello, B., Caffarra, S., Fariña, N., Duñabeitia, J., & Carreiras, M. (2021)
Reading without phonology: ERP evidence from skilled deaf readers of Spanish
DOI: 10.1038/s41598-021-84490-5

Error Rates

Fitted models:

```
> m0<-glmer(Err ~ Group + Type + (1 | sj),data=all, family=binomial)
> m1<-glmer(Err ~ Group * Type + (1 | sj),data=all, family=binomial)
> m2<-glmer(Err ~ Group * Type + (1 | sj) + (1 | item),data=all, family=binomial) # didn't converge
> m3<-glmer(Err ~ Group * Type + (1 + Type| sj),data=all, family=binomial) # didn't converge
```

Model comparison:

```
> anova(m0,m1)

   Df    AIC    BIC  logLik deviance  Chisq Chi Df Pr(>Chisq)
m0  5 2763.9 2797.7 -1376.9   2753.9
m1  7 2738.5 2785.9 -1362.3   2724.5 29.353      2  4.228e-07 ***
---
Signif. codes:  0 ‘***’ 0.001 ‘**’ 0.01 ‘*’ 0.05 ‘.’ 0.1 ‘ ’ 1
```

Best-fitting model summary:

```
Formula: Err ~ Group * Type + (1 | sj)

     AIC      BIC   logLik deviance df.resid
  2738.5   2785.9  -1362.3   2724.5     6393

Scaled residuals:
    Min      1Q  Median      3Q     Max
-0.6917 -0.2470 -0.2028 -0.1748  7.3802

Random effects:
 Groups Name        Variance Std.Dev.
 sj     (Intercept) 0.273    0.5225
Number of obs: 6400, groups:  sj, 40
```

DOI: 10.1038/s41598-021-84490-5

```
Fixed effects:
                Estimate Std. Error z value Pr(>|z|)    
(Intercept)     -3.23461    0.21301 -15.185  < 2e-16 ***
GroupH           0.08277    0.29568   0.280    0.780    
TypePH           0.22452    0.23503   0.955    0.339    
TypeW           -0.04756    0.21488  -0.221    0.825    
GroupH:TypePH    1.25181    0.30443   4.112 3.92e-05 ***
GroupH:TypeW     0.01849    0.29819   0.062    0.951    
---
Signif. codes:  0 ‘***’ 0.001 ‘**’ 0.01 ‘*’ 0.05 ‘.’ 0.1 ‘ ’ 1
```

Follow-up tests:

```
> lsmeans::lsmeans(m1, list(pairwise ~ Group*Type), adjust = "fdr")

Results are given on the logit (not the response) scale. 
Confidence level used: 0.95 

$`pairwise differences of contrast`
 contrast         estimate        SE     df z.ratio p.value
 D,PW - H,PW   -0.08276985 0.2956837  60.04  -0.280  0.8882
 D,PW - D,PH   -0.22452098 0.2350287  53.85  -0.955  0.7274
 D,PW - H,PH   -1.55910248 0.2607875  89.11  -5.978  <.0001
 D,PW - D,W     0.04755590 0.2148813  74.81   0.221  0.8882
 D,PW - H,W    -0.05370788 0.2713958 194.31  -0.198  0.8882
 H,PW - D,PH   -0.14175113 0.2861679  89.12  -0.495  0.8882
 H,PW - H,PH   -1.47633263 0.1934438 101.49  -7.632  <.0001
 H,PW - D,W     0.13032575 0.2699693 194.30   0.483  0.8882
 H,PW - H,W     0.02906197 0.2066741 154.62   0.141  0.8882
 D,PH - H,PH   -1.33458150 0.2499848  44.01  -5.339  <.0001
 D,PH - D,W     0.27207688 0.2017460  51.95   1.349  0.4437
 D,PH - H,W     0.17081311 0.2610450  85.81   0.654  0.8882
 H,PH - D,W     1.60665838 0.2312764  85.76   6.947  <.0001
 H,PH - H,W     1.50539460 0.1538603  94.00   9.784  <.0001
 D,W  - H,W    -0.10126378 0.2431836  70.91  -0.416  0.8882

Results are given on the log odds ratio (not the response) scale. 
P value adjustment: fdr method for 15 tests
```

Costello, B., Caffarra, S., Fariña, N., Duñabeitia, J., & Carreiras, M. (2021)
Reading without phonology: ERP evidence from skilled deaf readers of Spanish
DOI: [10.1038/s41598-021-84490-5](https://doi.org/10.1038/s41598-021-84490-5)

RTs

Fitted models:

```
> m0<-lmer(RTc ~ Group + Type + (1 | sj),data=all)
> m1<-lmer(RTc ~ Group * Type + (1 | sj),data=all)
> m2<-lmer(RTc ~ Group * Type + (1 | sj) + (1 | item),data=all)
> m3<-lmer(RTc ~ Group * Type + (1 + Type| sj) + (1 | item),data=all)
> m4<-lmer(RTc ~ Group * Type + (1 + Type| sj) + (1 + Group| item),data=all) # didn't converge
```

Model comparison:

```
> anova(m0,m1,m2,m3)

       Df   AIC   BIC logLik deviance   Chisq Chi Df Pr(>Chisq)
object  6 73113 73153 -36551    73101
..1     8 73084 73138 -36534    73068  33.035      2  6.708e-08 ***
..2     9 72906 72966 -36444    72888 179.979      1  < 2.2e-16 ***
..3    14 72807 72901 -36390    72779 109.239      5  < 2.2e-16 ***
---
Signif. codes:  0 '***' 0.001 '**' 0.01 '*' 0.05 '.' 0.1 ' ' 1
```

Best-fitting model summary:

```
> summary(m3)

Formula: RTc ~ Group * Type + (1 + Type | sj) + (1 | item)
REML criterion at convergence: 72738

Scaled residuals:
    Min      1Q  Median      3Q     Max
-7.1690 -0.5878 -0.1341  0.4439  5.0638

Random effects:
 Groups   Name        Variance Std.Dev. Corr
 item     (Intercept)  1211     34.80
 sj       (Intercept)  7564     86.97
          TypePH        691     26.29    0.34
          TypeW        1221     34.95   -0.43 -0.22
 Residual             13235    115.04
Number of obs: 5861, groups:  item, 240; sj, 40
```

DOI: 10.1038/s41598-021-84490-5

```
Fixed effects:
                 Estimate Std. Error        df t value Pr(>|t|)    
(Intercept)     654.05887   20.27613  40.91000  32.258  < 2e-16 ***
GroupH           73.65539   28.14272  37.97000   2.617 0.012658 *  
TypePH           -4.69554   10.03258  67.87000  -0.468 0.641264    
TypeW           -74.11995   10.86394  65.38000  -6.823 3.48e-09 ***
GroupH:TypePH    46.22370   11.98619  36.59000   3.856 0.000449 ***
GroupH:TypeW     -0.07314   13.25157  37.93000  -0.006 0.995625    
---
Signif. codes:  0 '***' 0.001 '**' 0.01 '*' 0.05 '.' 0.1 ' ' 1
```

<u>Follow-up tests:</u>

```
> lsmeans::lsmeans(m3, list(pairwise ~ Group*Type), adjust = "fdr")

Degrees-of-freedom method: satterthwaite 
Confidence level used: 0.95 

$`pairwise differences of contrast`
 contrast                estimate       SE    df t.ratio p.value
 D,PW - H,PW          -73.6553887 28.14272 37.97  -2.617  0.0173
 D,PW - D,PH            4.6955366 10.03258 67.87   0.468  0.7399
 D,PW - H,PH         -115.1835516 30.61232 43.56  -3.763  0.0009
 D,PW - D,W            74.1199546 10.86394 65.38   6.823  <.0001
 D,PW - H,W             0.5377108 27.26429 46.33   0.020  0.9843
 H,PW - D,PH           78.3509253 30.56447 43.29   2.563  0.0174
 H,PW - H,PH          -41.5281629 10.18518 72.00  -4.077  0.0002
 H,PW - D,W           147.7753433 27.26307 46.32   5.420  <.0001
 H,PW - H,W            74.1930995 10.87183 65.61   6.824  <.0001
 D,PH - H,PH         -119.8790882 31.91498 37.70  -3.756  0.0010
 D,PH - D,W            69.4244180 13.17145 52.52   5.271  <.0001
 D,PH - H,W            -4.1578258 29.24446 46.74  -0.142  0.9509
 H,PH - D,W           189.3035062 29.29333 47.05   6.462  <.0001
 H,PH - H,W           115.7212624 13.28857 54.39   8.708  <.0001
 D, W - H,W           -73.5822438 25.17909 37.98  -2.922  0.0087

P value adjustment: fdr method for 15 tests
```

Costello, B., Caffarra, S., Fariña, N., Duñabeitia, J., & Carreiras, M. (2021)
Reading without phonology: ERP evidence from skilled deaf readers of Spanish
DOI: [10.1038/s41598-021-84490-5](https://doi.org/10.1038/s41598-021-84490-5)

Anova with 40 items per each Type level

```
Factors (levels): Group (2) x Type (3) x Anteriority (3) x Laterality (3)
Time window: 300 - 500 ms
Univariate Type III Repeated-Measures ANOVA Assuming Sphericity

                                      SS num Df Error SS den Df      F    Pr(>F)
(Intercept)                      227.787      1   4155.4     38 2.0830 0.1571364
Group                             15.613      1   4155.4     38 0.1428 0.7076407
Type                             231.611      2   1020.7     76 8.6227 0.0004218 ***
Group:Type                        58.344      2   1020.7     76 2.1721 0.1209620
Anteriority                       84.169      2   1074.3     76 2.9771 0.0569110 .
Group:Anteriority                  1.282      2   1074.3     76 0.0453 0.9557096
Laterality                        70.614      2    599.4     76 4.4770 0.0145182 *
Group:Laterality                   3.582      2    599.4     76 0.2271 0.7973870
Type:Anteriority                  20.990      4     80.1    152 9.9623 3.468e-07 ***
Group:Type:Anteriority             6.838      4     80.1    152 3.2453 0.0137703 *
Type:Laterality                    5.293      4     70.2    152 2.8668 0.0251718 *
Group:Type:Laterality              0.946      4     70.2    152 0.5124 0.7266892
Anteriority:Laterality            12.517      4    335.3    152 1.4188 0.2303871
Group:Anteriority:Laterality       3.891      4    335.3    152 0.4411 0.7787802
Type:Anteriority:Laterality        0.332      8     43.4    304 0.2909 0.9687306
Group:Type:Anteriority:Laterality  0.752      8     43.4    304 0.6587 0.7277334
---
Signif. codes:  0 *** 0.001 ** 0.01 * 0.05 . 0.1   1


Mauchly Tests for Sphericity
                                  Test statistic p-value
Type                                     0.96467 0.51405
Group:Type                               0.96467 0.51405
Anteriority                              0.37390 0.00000
Group:Anteriority                        0.37390 0.00000
Laterality                               0.99013 0.83240
Group:Laterality                         0.99013 0.83240
Type:Anteriority                         0.33754 0.00001
Group:Type:Anteriority                   0.33754 0.00001
Type:Laterality                          0.83912 0.70099
Group:Type:Laterality                    0.83912 0.70099
Anteriority:Laterality                   0.59963 0.02877
Group:Anteriority:Laterality             0.59963 0.02877
Type:Anteriority:Laterality              0.14077 0.00059
Group:Type:Anteriority:Laterality        0.14077 0.00059
```

Costello, B., Caffarra, S., Fariña, N., Duñabeitia, J., & Carreiras, M. (2021)
Reading without phonology: ERP evidence from skilled deaf readers of Spanish
DOI: 10.1038/s41598-021-84490-5

```
Greenhouse-Geisser and Huynh-Feldt Corrections
 for Departure from Sphericity
                                  GG eps  Pr(>F[GG])
Type                              0.96588  0.0005025 ***
Group:Type                        0.96588  0.1229044
Anteriority                       0.61497  0.0832163 .
Group:Anteriority                 0.61497  0.8783856
Laterality                        0.99023  0.0148149 *
Group:Laterality                  0.99023  0.7952693
Type:Anteriority                  0.68677   1.49e-05 ***
Group:Type:Anteriority            0.68677  0.0285497 *
Type:Laterality                   0.90985  0.0296417 *
Group:Type:Laterality             0.90985  0.7095991
Anteriority:Laterality            0.80390  0.2388149
Group:Anteriority:Laterality      0.80390  0.7374464
Type:Anteriority:Laterality       0.70563  0.9335770
Group:Type:Anteriority:Laterality 0.70563  0.6737356
---
Signif. codes:  0 *** 0.001 ** 0.01 * 0.05 . 0.1   1
```

Follow up t-test for Group x Type x Anteriority

Deaf

Averages (μV)

```
     Anterior    Central Posterior
PH  0.4042012  0.2302393 0.7010564
PW -0.3362148 -0.3253005 0.5694203
W   0.8863827  1.3730351 2.3425231
```

Type contrasts in each level of Anteriority

Anterior

```
              t df       p p.hocberg
PH-PW  1.794489 19 0.08866   0.35464
PH-W  -1.276006 19 0.21733   0.52664
PW-W  -2.761769 19 0.01241   0.07446
```

DOI: 10.1038/s41598-021-84490-5

Central

	t	df	p	p.hocberg
PH-PW	1.152729	19	0.26332	0.52664
PH-W	-2.341489	19	0.03026	0.15130
PW-W	-3.232373	19	0.00438	0.03080

Posterior

	t	df	p	p.hocberg
PH-PW	0.2915284	19	0.77381	0.77381
PH-W	-3.2305091	19	0.00440	0.03080
PW-W	-3.8776317	19	0.00101	0.00909

<u>Hearing</u>

Averages (μV)

	Anterior	Central	Posterior
PH	0.3852590	0.5626121	1.2803667
PW	-0.6473849	-0.4971468	0.5053274
W	0.2241816	0.5315174	1.3363927

<u>Type contrasts in each level of Anteriority</u>

Anterior

	t	df	p	p.hocberg
PH-PW	4.0704316	19	0.00065	0.00585
PH-W	0.4224456	19	0.67744	0.92733
PW-W	-2.0427641	19	0.05520	0.22080

Central

	t	df	p	p.hocberg
PH-PW	3.83718961	19	0.00111	0.00888
PH-W	0.09242841	19	0.92733	0.92733
PW-W	-2.67211812	19	0.01507	0.07535

Posterior

	t	df	p	p.hocberg
PH-PW	3.0843011	19	0.00610	0.04270
PH-W	-0.1872354	19	0.85346	0.92733
PW-W	-2.8023541	19	0.01137	0.06822
